# Recurrences and progression following microsurgery of vestibular schwannoma

**DOI:** 10.3389/fsurg.2023.1216093

**Published:** 2023-06-21

**Authors:** Maximilian Scheer, Sebastian Simmermacher, Julian Prell, Sandra Leisz, Christian Scheller, Christian Mawrin, Christian Strauss, Stefan Rampp

**Affiliations:** ^1^ Department of Neurosurgery, Medical Faculty, University Hospital Halle, Martin-Luther-University Halle-Wittenberg, Halle (Saale), Germany; ^2^ Department of Neuropathology, University Magdeburg, Magdeburg, Germany

**Keywords:** capsule residual, extent of resection, gross total resection, near total resection, subtotal resection, tumor recurrence, tumor progression

## Abstract

**Background:**

The treatment approach of vestibular schwannoma (VS) has seen a change in recent years, with a trend away from radical surgery towards preservation of cranial nerve function. A recent study reported recurrences as long as 20 years after complete removal of VS.

**Objective:**

To report the risk of recurrence and progression in our patient population the authors retrospectively reviewed outcomes of patients.

**Methods:**

Cases with unilateral VS who had undergone primary microsurgery via retrosigmoidal approach between 1995 and 2021 were investigated. Complete tumor removal was defined as gross total resection (GTR), a capsular remnant was categorized as near total resection (NTR) and residual tumor was designated as subtotal resection (STR). The primary endpoint was radiological recurrence-free survival.

**Results:**

386 patients fulfilled the inclusion criteria of the study and were evaluated. GTR was achieved in 284 patients (73.6%), NTR was achieved in 63 patients (10.1%) and STR was present in 39 patients (16.3%). A total of 28 patients experienced recurrences with significant differences in the three subgroups. The strongest predictor of recurrence was the extent of resection, with patients who underwent STR having an almost 10-fold higher risk of recurrence and patients who had undergone NTR having an almost 3-fold higher risk than those treated with GTR. More than 20% of recurrences (6/28) occured after more than 5 years.

**Conclusion:**

The degree of resection is an important guide to the interval of follow-up, but long-term follow-up should be considered also in the case of GTR. The majority of recurrences occurs after 3–5 years. Nevertheless, a follow-up of at least 10 years should be carried out.

## 1. Introduction

Vestibular schwannomas (VS) are benign tumors of schwann cells of the vestibulocochlear nerve. They represent 85% of tumors of the cerebellopontine angle. Although benign, size growth can cause diverse and severe deficits (1). Treatment should be individualized to the patient to achieve tumor control with the best possible functional outcome. The options range from wait and see, radiotherapy to microsurgery (2–4).

In recent years, a paradigm shift has been observed in treatment. Instead of radical surgery with increased risk of facial palsy and hearing loss, subtotal resection followed by radiotherapy is often favored, to achieve a better functional outcome (5–7). This trend is also reflected in the current European Association of Neuro-Oncology (EANO) guideline (2).

Depending on the surgeon's experience, tumor size, existing deficits and patient's choice, different approaches can be used for surgery (8). In general, neurophysiological monitoring is essential during these procedures to achieve the best possible functional outcome (9–11).

Some authors describe the tumor capsule as a good dissection plane for tumor removal. Leaving the capsule in place is said to be associated with a low risk of recurrence (12). In addition, the extent of resection correlates with risk of recurrence as well as the functional outcome: Gross total resection (GTR) has a low risk of recurrence but is associated with worse functional outcome whereas a large tumor remnant in subtotal resection is associated with a high probability of recurrence (13, 14). However, some studies showed that near total resection (e.g., leaving capsule remnant) may achieve similar results to GTR (14).

One argument for radical surgery is the best long-term outcomes. In the literature, there is however a great variability regarding recurrence rate after GTR with a wide range from almost zero to just under 10%, but these studies usually have a short follow-up time of fewer than 5 years (15–18).

Furthermore, there is no consistent recommendation for follow-up time after VS surgery and the growth rate is unpredictable (4, 17). Nakatomi et al. were able to show that recurrences can occur even 20 years after GTR. In their study, they show a recurrence rate of 13% even with GTR (19).

We took this study as motivation to reevaluate our patients. Based on the mentioned finding on recurrences even decades after GTR, we had implemented additional MRI scans in our collective and could thus generate data on long-term results.

## 2. Methods

### 2.1. Population

We retrospectively reviewed the records of all patients with sporadic VS who had undergone primary microsurgical resection via retrosigmoidal approach between 1995 and 2021. Neurophysiological monitoring was used as standard and all patients were operated on by the same surgeon. Patients with neurofibromatosis type II/bilateral acoustic neuromas, previous irradiation or previous external surgery were not included. The inclusion criterion was the availability of at least one postoperative imaging investigation.

### 2.2. Data collection

Patient-related data such as age, gender and tumor localization were recorded. Tumor sizes were classified according to the Koos classification (1–4) (20). Facial function was evaluated preoperatively and before discharge according to the House Brackmann classification (HB) (21) and the hearing quality was evaluated according to the American Academy of Otolaryngology—Head and Neck Surgery (AAO-HNS) classification (A–D) (22). In addition to categories A–D, category E was added in the case of complete deafness. Both were recorded pre- and postoperatively. In addition, it was evaluated whether the auditory nerve could be preserved anatomically. The extent of resection was divided into gross total resection (GTR), near total resection (NTR) and subtotal resection (STR) according to the surgeon's intraoperative impression. NTR is defined as only capsule remnant. The primary endpoint was radiological recurrence-free survival in months, defined as the period from the date of initial surgery to the date of radiological diagnosis of recurrence or progression of residual tumor.

### 2.3. Evaluation of postoperative MRI

Postoperative imaging was evaluated by an experienced radiologist as well as the surgeon. For GTR, a new contrast enhancement was defined as recurrence. In case of NTR or STR, the first MRI after surgery served as new baseline. An increase of contrast enhancement was defined as progression.

### 2.4. Statistical analysis

Recurrence-free survival was evaluated using Kaplan-Meier survival statistics utilizing a log-rank test for comparison of group differences. Duration of follow-up was calculated from the date of surgery to the date of radiological diagnosis of recurrence or last radiological follow-up. Associations between the studied features and tumor recurrence were evaluated. For comparison of frequencies, chi-square- or the exact Fisher-test were used. Group differences in regard to age, tumor size, pre- and postoperative facials nerve function and hearing were evaluated using ANOVA- and Kruskal-Wallis-test. *p*-values <0.05 were considered statistically significant.

## 3. Results

### 3.1. Patient characteristics

In total, data from 547 patients were analyzed. Due to lack of postoperative imaging, preoperative radiotherapy, previous external surgery, or neurofibromatosis type II, 161 patients were excluded. This yielded a total of 386 patients which were included in this study. Among these, there were 240 women (62.2%) and the median age was 54 years (range 21–80 years). The mean age in the GTR group was 49.4 (±12.0) years, 54.2 (±14.6) years in the NTR group and 62.7 (±13.0) years in the STR group (*p* < 0.0001).

One hundred and eighty-nine VS were localized on the left side (48.9%; *p* = 0.25). Preoperatively, a median hearing class A was present in the GTR group, C in the NTR and D in the STR group respectively (*p* < 0.0001). Nearly all patients showed normal facial function (270 HB I, 114 HB II), with a mean HB I in all groups (*p* = 0.0003). Baseline features are summarized in Table 1.

**Table 1:** Summary of features of 386 VS patients.

	GTR	NTR	STR	*p*-value	Statistical test
Number	284 (73.6%)	63 (16.3%)	39 (10.1%)		
Sex					
Male	109 (74.7%)	24 (16.4%)	13 (8.9%)	0.83	Chi-squared
Female	175 (72.9%)	39 (16.3%)	26 (10.8%)		
Age	49.4 (±12.0)	54.2 (±14.6)	62.7 (±13.0)	<0.0001	ANOVA
Koos (median, 1.–3. quartile)	3 (2–3)	4 (3–4)	4 (3–4)	<0.0001	Kruskal-Wallis
Koos 1	30	0	0		
Koos 2	100	3	1		
Koos 3	114	17	10		
Koos 4	40	43	28		
Side					
Left	132 (69.8%)	36 (19.0%)	21 (11.1%)	0.25	Chi-squared
Right	152 (77.2%)	27 (13.7%)	18 (9.1%)		
Preop facial nerve function (HB, median, 1.–3. quartile)	I (I–I)	I (I–II)	I (I–II)	0.0003	Kruskal-Wallis
Preop AAO-HNS (median, 1.–3. quartile)	A (A–C)	C (B–D)	D (B–E)	<0.0001	Kruskal-Wallis

AAO-HNS, American Academy of Otolaryngology—Head and Neck Surgery; HB, House Brackmann; GTR, gross total resection; NTR, near total resection; STR, subtotal resection.

### 3.2. Tumor characteristics and operative details

At the time of surgery, median tumor size was Koos 3 (range 1–4). Regarding the subgroups, we found significantly different tumor sizes: median tumor size Koos 3 in the GTR group and Koos 4 for the NTR and STR group (*p* < 0.0001). In all cases, histopathological examination revealed a benign schwannoma without atypia. GTR was achieved in 284 cases (73.6%). 39 patients underwent STR (10.1%) and in 63 patients NTR (16.3%) was achieved. In 211 cases, the cochlear nerve was anatomically preserved (54.7%).

### 3.3. Facial nerve function, hearing preservation, tumor control

**Figure 1:**
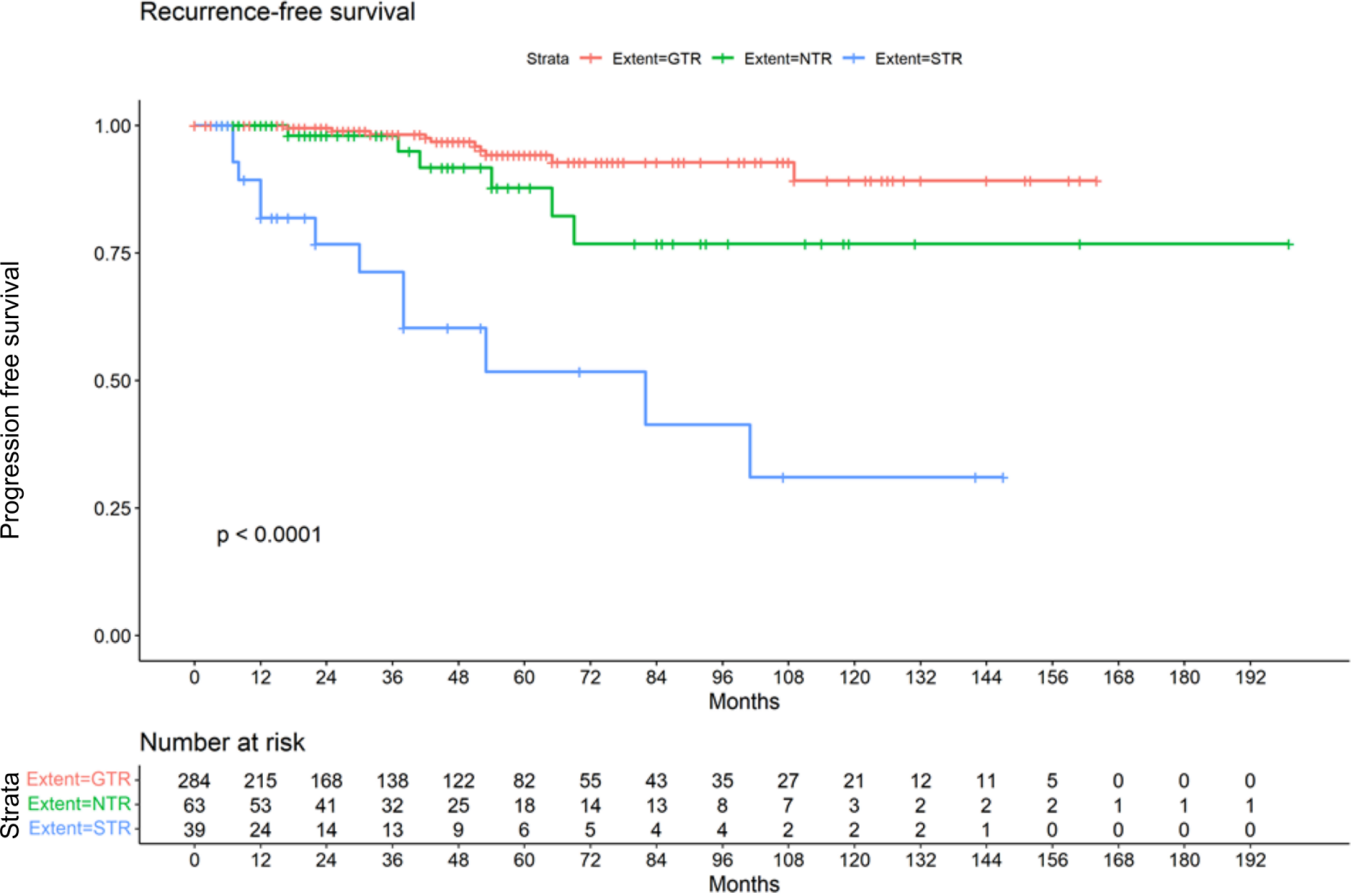
Kaplan-Meier Plot demonstrating recurrence-free survival by GTR, NTR and STR.

Postoperatively 200 patients (51.8%) showed normal facial nerve function (HB I-II). A median HB II was found in the GTR and STR group. In the group that underwent NTR a median HB IV was present (*p* < 0.0001). The median hearing class postoperatively was E in all subgroups, while the range was C-E in the GTR group. All patients in the STR and NTR groups showed a hearing class E postoperatively (*p* < 0.0001). In the GTR, cochlear nerve could be preserverd in 181 cases (63.7%). In the NTR group, preservation was possible in 14 cases (22.2%) and in 16 cases (41.0%) of the STR group (*p* < 0.0001). Radiological follow-up was on average 44.3 months (range 2–199 months). The longest follow-up time with a mean of 48.5 (±41.2) months was found in the NTR group. A mean follow-up of 45.4 months (±39.8) was present in the GTR group. The shortest follow-up time with a mean of 34.4 (±38.3) months belonged to the STR group. The differences were however not significant (*p* = 0.20) (Table 2). Of 386 patients, 28 suffered a recurrence or progression (7.3%), including 19 women (67.9%). Among the 284 cases in which a GTR was achieved, 10 developed a recurrence (3.5%). Of the 63 cases in which NTR was achieved, 6 developed a recurrence (9.5%). This affected 4 women and 2 men. In total, there were 12 cases with progression in the group that underwent STR (30.8%). Here, a distribution of 10:2 (female: male) was found. However, gender was not significantly associated with recurrence (*p* = 0.29). A summary of postoperative results in all patients can be found in Table 2. The Kaplan-Meier plot shows the recurrence-free survival of the 3 subgroups (Figure 1).

**Table 2:** Summary of postoperative results in all patients.

	GTR	NTR	STR	*p*-value	Test
Recurrences total	10/284 (3.5%)	6/63 (9.5%)	12/39 (30.8%)	<0.0001	Fisher's Exact Test
Follow-up (months, SD)	45.4 (±39.8)	48.5 (±41.2)	34.4 (±38.3)	0.20	ANOVA
Postop facial nerve function (HB, median, 1.–3. quartile)	II (II–III)	IV (III–IV)	II (II–III)	<0.0001	Fisher's Exact Test
Postop AAO-HNS (median, 1.–3. quartile)	E (C–E)	E (E–E)	E (E–E)	<0.0001	Fisher's Exact Test
Preserved cochlear nerve	181 (63.7%)	14 (22.2%)	16 (41.0%)	<0.0001	Fisher's Exact Test

AAO-HNS, American Academy of Otolaryngology—Head and Neck Surgery; HB, House Brackmann; SD, standard deviation; GTR, gross total resection; NTR, near total resection; STR, subtotal resection.

### 3.4. Association between baseline features and recurrence

In total 28 patients experienced recurrence respectively progression. The strongest predictor was extent of resection (Cox regression with factors resection extent and tumor size). Compared to the risk of recurrence in the GTR group (3.5%; *p* < 0.0001) NTR was associated with a 3 times higher risk of recurrence (9.5%) and patients who received STR showed a 10 times higher risk (30.8%). Tumor size remained nonsignificant (*p* = 0.37). The gender distribution in the GTR group was equal (5 female, 5 male). While we found more women in the NTR (66.7%) and STR group (83.3%; *p* = 0.29). Time to recurrence respectively progression was shortest in the STR group with a median of 26 months (10–45.5, 1st/3rd quartile). Median time to recurrence was 47 months (32–53) in the GTR and 47.5 (37–65) in the NTR group respectively (*p* < 0.0001, ANOVA). A median HB II was found in the GTR. In the STR group we found a median HB II–III. In the group that underwent NTR a median HB III was present (*p* = 0.43). Postoperative hearing was not significantly different in the subgroups (GTR with C/D; NTR and STR E; *p* = 0.038). However, preservation of the cochlear nerve varied strongly in the subgroups. This preservation was possible in 90% of patients in the GTR group, who experienced recurrence, whereas it was preserved in only 33.3% of patients in the NTR and STR group who experienced recurrence (*p* = 0.015). A summary of patients with recurrence can be found in Table 3.

**Table 3:** Summary of postoperative results in patients with recurrence.

	GTR	NTR	STR	*p*-value	Test
Time to Recurrence (months, median, 1st/3rd quartile)	47 (32–53)	47.5 (37–65)	26 (10–45.5)	<0.0001	ANOVA
Postop facial nerve function (HB, median, 1.–3. quartile)	II (I–IV)	III (II–IV)	II-III (II–III)	0.43	Fisher's Exact Test
Postop AAO-HNS (median, 1.–3. quartile)	C/D (B–E)	E (D–E)	E (E–E)	0.038	Fisher's Exact Test
Female	5/10 (50%)	4/6 (66.7%)	10/12 (83.3%)	0.29	Fisher's Exact Test
Preserved cochlear nerve	9/10 (90%)	2/6 (33.3%)	4/12 (33.3%)	0.015	Fisher's Exact Test

AAO-HNS, American Academy of Otolaryngology—Head and Neck Surgery; HB, House Brackmann; SD, standard deviation; GTR, gross total resection; NTR, near total resection; STR, subtotal resection.

### 3.5. Treatment strategies following radiological recurrence

In the majority of the cases with recurrence (19/28 patients), radiotherapy was subsequently performed. In 6 cases, a second surgery was performed. In 3 patients, no further treatment was performed so far due to the small size, however regular MRI controls are performed.

## 4. Discussion

In this study, we compare the outcome of VS patients according to the extent of resection. We demonstrated that GTR is associated with the lowest risk of recurrence (3.5%). In this group, tumors were smallest (Koos 3), patients were younger (49.4 years), and facial nerve function was best postoperatively (HB II), which again can be explained by tumor size. NTR was associated with an increased risk of recurrence (9.5%). The tumors were larger compared with the GTR group (Koos 4), and the facial nerve function was worse postoperatively (HB IV). This observation can be explained by the fact that the patients were significantly younger than in the STR group (54.2 vs. 62.7 years) and the surgeon wanted to operate as radically as possible to reduce the risk of recurrence. Therefore, the lowest rate of anatomically preserved cochlear nerves (22.2%) is also found in this group. In the STR, the patients were the oldest, and the tumors were the largest, as in the NTR group, with a Koos 4. Due to the age, less radical surgery was performed, so that a good functional outcome (HB II) is also found here. Here nearly one third of patients experienced recurrence (30.8%).

Follow-up after microsurgery of VS is managed very differently and shows great variability. Unfortunately, there are no uniform recommendations for the follow-up time and the span ranges from 3 to 10 years (2, 23). Moreover, many studies have an average follow-up of fewer than 5 years (24, 25). However, most authors now recommend a follow-up of 10 years (3, 4, 17). The study by Nakatomi et al. showing recurrences even decades after GTR of VS was a motivation to reexamine our collective (19). In contrast to this study, we have added NTR as a further category in addition to GTR and STR.

The risk of recurrence after GTR of a VS varies widely in the literature between 0.3% and 9.2%. Most authors report a 2%–4% risk of recurrence (14–16, 25). With 13.1% only the study by Nakatomi et al. shows a much higher frequency, which was explained by the very long follow-up time up to 20 years (19). In our cohort, the risk of recurrence after GTR was 3.5%, which is comparable to the previously mentioned studies (14, 25). The lower recurrence rate is likely due to the shorter follow-up times. This is reflected by the mean times to recurrence: Nakatomi et al. report a mean time to recurrence of 90 months after GTR with a range up to 20 years (19). In comparison, we found a mean time to recurrence of 48.9 and 47.2 months in the GTR and NTR group with a wide range of up to 102 months and the longest follow-up of 16 years.

The surgical aim of NTR is often considered to ensure the integrity of the nervous structures, and it is known to be associated with a very low risk of recurrence. In a large study of Carlson et al., GTR, NTR and STR after microsurgery with an average follow-up of 42 months of VS were compared. Here, no significant difference was found between GTR and NTR (25). This is in line with the results of Seol et al., who also compared GTR, NTR and STR with an average follow-up of 55 months and also could not find any differences in the first two groups (14). In addition, Cerulla et al. reported that leaving coagulated tumor bits do not affect the risk of recurrence after microsurgery of VS (26). In contrast to these studies, we found an increased risk of recurrence between GTR and NTR (3.52% vs. 9.52%) in our cohort. This effect might be explained by the assessment of the surgeon as we have seen similar follow-up intervals. A study from Godofroy et al. could demonstrate, that the intraoperative impression of tumor removal in VS surgery is imperfect so that a larger residual tumor can be present when the intraoperative impression was NTR (27). Besides that, another investigation could show that the tumor capsule contains viable neoplastic cells, which, of course, can lead to recurrence in the long term (28).

The fact that subtotal resection is associated with an increased risk of recurrence has already been described several times. Beshears et al. reported that 30% of patients who underwent STR of VS suffered recurrences and there was a strong correlation between remaining tumor size and recurrence risk (13). Also in the study of Seol et al. there was a high rate of recurrence after STR in nearly one third of the patients (14). This is in line with our results, where 30.7% of all patients who underwent STR experienced recurrence. Based on their long-term results, Nakatomi et al. (19) suggest, that if the follow-up is long enough, the majority of patients treated with STR will experience recurrence. Given the shape of the Kaplan-Meier curve of our cohort, this statement also seems conceivable for our collective. Summarizing all recurrences, we find that the majority of these events occur within the first 96 months but in line with the suggestion of Nakatomi and collegues also in our collective some recurrences were detected later. The fact, that the mean follow-up time of 45.4 months in the GTR group was lower than the mean time to recurrence (48.9 months) implies that asymptomatic recurrences might be missed and the number of observed late recurrences may in fact be an underestimation. Recent work on predicting VS recurrence and postoperative outcome using artificial neural networks supported our findings. The degree of resection correlated with the risk of recurrence and larger tumours were associated with poorer postoperative outcome (29, 30).

In our three subgroups, we found differences in follow-up time and age. GTR was more common in younger patients. While older patients tended to undergo STR or NTR, as the risk of experiencing recurrence due to life expectancy appears to be lower. In addition, the follow-up was shortest in the STR group, which can be explained by the early occurrence of recurrences.

In recent years, the treatment approach of VS has undergone a change and is moving away from radical surgery. According to EANO guidelines small, incidental findings (Koos 1 or 2) should be observed or alternatively irradiated. For small, symptomatic findings (Koos 1 or 2), stereotactic radiosurgery (SRS) is preferred to microsurgery because of better functional outcomes. Larger findings (Koos 3 or 4) should rather be treated surgically to relieve the surrounding tissue. Here, mass reduction followed by SRS of the residual tumor should be considered as a valid option (2). The combination of STR followed by SRS was shown to have very good results in a study regarding facial function with 95% of HB I–II (6). However, reliable long-term data of this combination are needed to be able to assess the risk of recurrence. Furthermore, severe complications such as malignant transformation or demyelination of the surrounding neural structures are rare but can also occur and should be addressed when counseling patients (31, 32).

As we could show in our study, good functional outcomes can also be achieved in GTR, which is associated with a low risk of recurrence. Based on our data, we believe that the risk of recurrence, even over a long period, is underestimated and we suspect a high number of asymptomatic findings in our collective.

Although we have contacted our patients again and recommended new MRI scans, the long-term data are weak as we have data from 26 patients after 10 years. A follow-up of 5 years seems insufficient because even with the said limitations, more than 20% of recurrences (6/28) occur after more than 5 years. Further limitations to this weak long-term data are the retrospective study design and that the data are based on the subjective assessment of the surgeon regarding the extent of resection.

## 5. Conclusion

In the current study, we analyzed outcomes after microsurgery of VS via retrosigmoidal approach of 386 patients. The degree of resection is an important guide regarding the recommended follow-up interval. Nevertheless, long-term follow-up should also be considered even in case of GTR. An inconspicuous MRI finding after 5 years suggests a false sense of security and asymptomatic findings may be missed. This study also shows that GTR can be associated with good functional outcomes and a low risk of recurrence.

## Data Availability

The raw data supporting the conclusions of this article will be made available by the authors, without undue reservation.
